# Better CRT Response in Patients Who Underwent Atrioventricular Node Ablation or Upgrade From Pacemaker: A Nomogram to Predict CRT Response

**DOI:** 10.3389/fcvm.2021.760195

**Published:** 2021-11-01

**Authors:** Pei-Lin Xiao, Cheng Cai, Pei Zhang, Jie Han, Siva K. Mulpuru, Abhishek J. Deshmukh, Yue-Hui Yin, Yong-Mei Cha

**Affiliations:** ^1^Department of Cardiology, The Second Affiliated Hospital of Chongqing Medical University, Chongqing, China; ^2^Department of Cardiology, The First Affiliated Hospital of Nanjing Medical University, Nanjing, China; ^3^Department of Cardiology, Sir Run Run Shaw Hospital, The First Affiliated Hospital of Zhejiang University, Hangzhou, China; ^4^Department of Cardiology and Atrial Fibrillation Center, The First Affiliated Hospital of Zhejiang University, Hangzhou, China; ^5^Department of Cardiovascular Medicine, Mayo Clinic, Rochester, MN, United States

**Keywords:** cardiac resynchronization therapy, left bundle-branch block, atrioventricular node ablation, nomogram, left ventricular ejection fraction (LVEF)

## Abstract

**Background:** Response rates for cardiac resynchronization therapy (CRT) in patients without intrinsic left bundle-branch block (LBBB) morphology are poor.

**Objective:** We sought to develop a nomogram model to predict response to CRT in patients without intrinsic LBBB.

**Methods:** We searched electronic health records for patients without intrinsic LBBB who underwent CRT at Mayo Clinic. Logistic regression and Cox proportional hazards regression analysis were performed for the odds of response to CRT and risk of death, respectively. Results were used to develop the nomogram model.

**Results:** 761 patients without intrinsic LBBB were identified. Six months after CRT, 47.8% of patients demonstrated improvement of left ventricular ejection fraction by more than 5%. The 1-, 3-, and 5-year survival rates were 95.9, 82.4, and 66.70%, respectively. Patients with CRT upgrade from pacemaker [odds ratio (OR), 1.67 (95% CI, 1.05–2.66)] or atrioventricular node (AVN) ablation [OR, 1.69 (95% CI, 1.09–2.64)] had a greater odds of CRT response than those patients who had new implant, or who did not undergo AVN ablation. Patients with right bundle-branch block had a low response rate (39.2%). Patients undergoing AVN ablation had a lower mortality rate than those without ablation [hazard ratio, 0.65 (95% CI, 0.46–0.91)]. Eight clinical variables were automatically selected to build a nomogram model and predict CRT response. The model had an area under the receiver operating characteristic curve of 0.71 (95% CI, 0.63–0.78).

**Conclusions:** Among patients without intrinsic LBBB undergoing CRT, upgrade from pacemaker and AVN ablation were favorable factors in achieving CRT response and better long-term outcomes.

## Introduction

Cardiac resynchronization therapy (CRT) is an effective therapy for patients with heart failure with reduced ejection fraction (HFrEF), left bundle-branch block (LBBB), and/or a wide QRS complex. Randomized clinical trials (RCTs) have shown CRT to be beneficial in improving heart failure (HF) symptoms, left ventricular ejection fraction (LVEF), quality of life, and survival (1–6).

Aside from QRS duration, intrinsic LBBB is generally considered an important determinant of CRT response. The current American College of Cardiology/American Heart Association/Heart Rhythm Society (ACC/AHA/HRS) and European Society of Cardiology guidelines provide the strongest recommendations for the use of CRT in patients with typical LBBB pattern and wide QRS, who benefit most from CRT (7–9). However, nearly 20% to 50% of CRT recipients do not have a good outcome after CRT (10, 11). Conversely, some patients without intrinsic LBBB may have HF improvement (12, 13). These patients usually refers to right bundle-branch block (RBBB), intraventricular conduction delay (IVCD), and predominantly ventricular paced rhythm with non-physiologic depolarization pattern (14, 15). Some *post-hoc* analyses of landmark RCTs have shown a wide range of CRT response for HFrEF patients without intrinsic LBBB (1, 2, 16–18). The REVERSE (REsynchronization reVErses Remodeling in Systolic Left vEntricular Dysfunction) trial showed that some patients with HFrEF and RBBB derived significant improvement from CRT (18). However, meta-analysis suggested that HFrEF patients without LBBB morphology do not have improvement of mortality or HF hospitalization rates after CRT (19). Therefore, it may be helpful to build a specific and practical predictive model that incorporates variables from a real-word clinical database of CRT recipients without intrinsic LBBB. This study aimed to develop an evidence-based nomogram and use it to predict CRT responders in patients without intrinsic LBBB morphology (20).

## Methods

### Study Population and Data Source

The study was approved by the Mayo Clinic Institutional Review Board. Between 2002 and 2017, a prospectively designed CRT database collected data from the electronic health record of patients who underwent CRT (pacemaker or implantable cardioverter-defibrillator [ICD]) implantation at Mayo Clinic, Rochester, Minnesota. For this study, we queried the CRT database for the records of patients who underwent successful CRT implantation; for whom preoperative electrocardiography (ECG) did not indicate typical intrinsic LBBB morphology, which was defined as follows: the QRS duration ≥120 ms, QS or rS pattern on V1, Notch/slurred R-wave on I, aVL, V5, V6, and absent Q-wave on V5, V6 (21); and who had available LVEF data at baseline and at 6-month follow-up. Patients who underwent replacement of CRT pulse generator, lead revision or His-Purkinje conduction system pacing were excluded.

### Baseline Clinical Data Collection and Preparation

All baseline clinical data and patient demographics were extracted from our database. The missing data points were rechecked through chart review. The key variables from ECG and LVEF were validated by 2 independent investigators (P.-L.X. and J.H.).

### Electrocardiography

All available standard 12-lead ECG results before CRT implantation were assessed by chart review. LBBB, RBBB, and IVCD were defined according to the criteria approved by the World Health Organization and 2013ESC guidelines (21, 22).

### Echocardiography

All echocardiography parameters were collected by reviewing the echocardiography report. LVEF, left ventricular end-diastolic dimension (LVEDD), and pulmonary artery systolic pressure were collected.

### Outcome Measures

The primary endpoint of the study was all-cause death. In addition, we also assessed the benefit of CRT by assessing echocardiographic response at 6-month follow-up. CRT response was defined as an improvement of LVEF by >5% in absolute value at 6-month follow-up after CRT.

### Statistical Analysis

Categorical variables were presented as counts and percentages, and continuous variables were reported as mean (SD) or median (interquartile range). Univariable and multivariable logistic regression analysis and Cox proportional hazards regression analysis, respectively, were performed to evaluate the effects of potential predictors on CRT response [reported as odds ratio (OR)] and overall survival [reported as hazard ratio (HR)]. Unadjusted overall survival was estimated with the Kaplan-Meier method, and groups were compared by using the log-rank test. Interaction and stratified analyses were performed for the predictor that had significant interaction with other variables.

For prediction model development and evaluation, we randomly partitioned the study population into training (70%) and validation (30%) sets using statistical software (R version 3.4.3). Backward stepwise selection with the Akaike information criterion was used to select variables for the multivariable logistic regression model. Nomograms were formulated incorporating selected variables to predict the probability of response after CRT implantation using statistical software (rms in R, version 3.4.3; http://www.r-project.org) (20).

The predictive performance of the nomogram included discrimination ability and calibration. The area under the receiver operating characteristic curve was calculated to measure predictive accuracy for individual outcomes. Calibration with 1,000 bootstrap samples was measured by plotting the predicted frequencies against the observed probabilities to determine the prediction capability of the nomogram (23). Statistical analyses were performed with R software, version 3.4.3. Two-sided *P*-values <0.05 were considered statistically significant.

## Results

### Baseline Demographics and Clinical Characteristics

Our search of the database identified 761 patients who received CRT with an ICD (83.6%) or pacemaker (16.4%) and met the inclusion and exclusion criteria from 2002 to 2017. The mean (SD) age was 69 (12) years, and 602 (79%) of the patients were men (Table 1). QRS morphology at baseline was paced in 57.4%, RBBB in 14.7%, and IVCD in 19.3%; 8.5% had normal QRS morphology with atrial fibrillation and were undergoing atrioventricular node (AVN) ablation. A total of 450 patients (59.1%) had atrial fibrillation, of whom 131 (29.1%) had AVN ablation. The mean (SD) LVEF was 29.2% (9.9%). Most of the patients (*n* = 535, 70.3%) were undergoing CRT upgrade from a pacemaker (*n* = 281) or ICD (*n* = 254), and the rest (226, 29.7%) had a de novo CRT implant.

**Table 1:** Patient characteristics.

**Characteristic**	**Value^a^ (*N* = 761)**
Age, y	69.3 (12.3)
Men	602 (79.1%)
Body mass index	29.8 (6.1)
**Comorbid conditions**	
Hypertension	334 (43.9%)
Coronary artery disease	404 (53.1%)
Diabetes	190 (25.0%)
Chronic kidney disease	235 (30.9%)
Ischemic cardiomyopathy	362 (47.6%)
Atrial fibrillation	450 (59.1%)
High-degree atrioventricular block^b^	293 (38.5%)
AVN ablation	131 (17.2%)
Prior AVN ablation + CRT upgrade	87 (11.4%)
AVN ablation + de novo CRT	44 (5.8%)
NYHA class	2.78 (0.61)
Pacemaker dependent	312 (41.0%)
QRS duration, ms	167.6 (36.9)
**QRS duration**	
<150 ms	240 (31.5%)
≥150 ms	521 (68.5%)
**QRS morphology**	
Paced QRS	437 (57.4%)
RBBB	112 (14.7%)
IVCD	147 (19.3%)
Normal	65 (8.5%)
LVEF, %	29.21 (9.90)
LVEDD, mm	62.23 (8.69)
PASP, mm Hg	43.80 (14.63)
**Medications**	
Digoxin	225 (29.6%)
β-Blocker	644 (84.6%)
RAS inhibitor	540 (71.0%)
Spironolactone	172 (22.60%)
Furosemide	444 (58.3%)
**Laboratory values**	
Hemoglobin, g/dL	13.00 (1.87)
Creatinine, mg/dL	1.37 (0.57)
**Procedures**	
**Device placed**	
ICD	636 (83.6%)
PM	125 (16.4%)
Upgrade from PM or ICD	535 (70.3%)

*AVN, atrioventricular node; CRT, cardiac resynchronization therapy; ICD, implantable cardioverter-defibrillator; IVCD, intraventricular conduction delay; LVEDD, left ventricular end-diastolic dimension; LVEF, left ventricular ejection fraction; NYHA, New York Heart Association; PASP, pulmonary artery systolic pressure; PM, pacemaker; RAS, renin-angiotensin system; RBBB, right bundle-branch block*.

a *Values are mean (SD) or No. of patients (%)*.

b *High-degree atrioventricular block was defined as second- and third-degree atrioventricular block*.

### CRT Response and Independent Predictors

**Figure 1:**
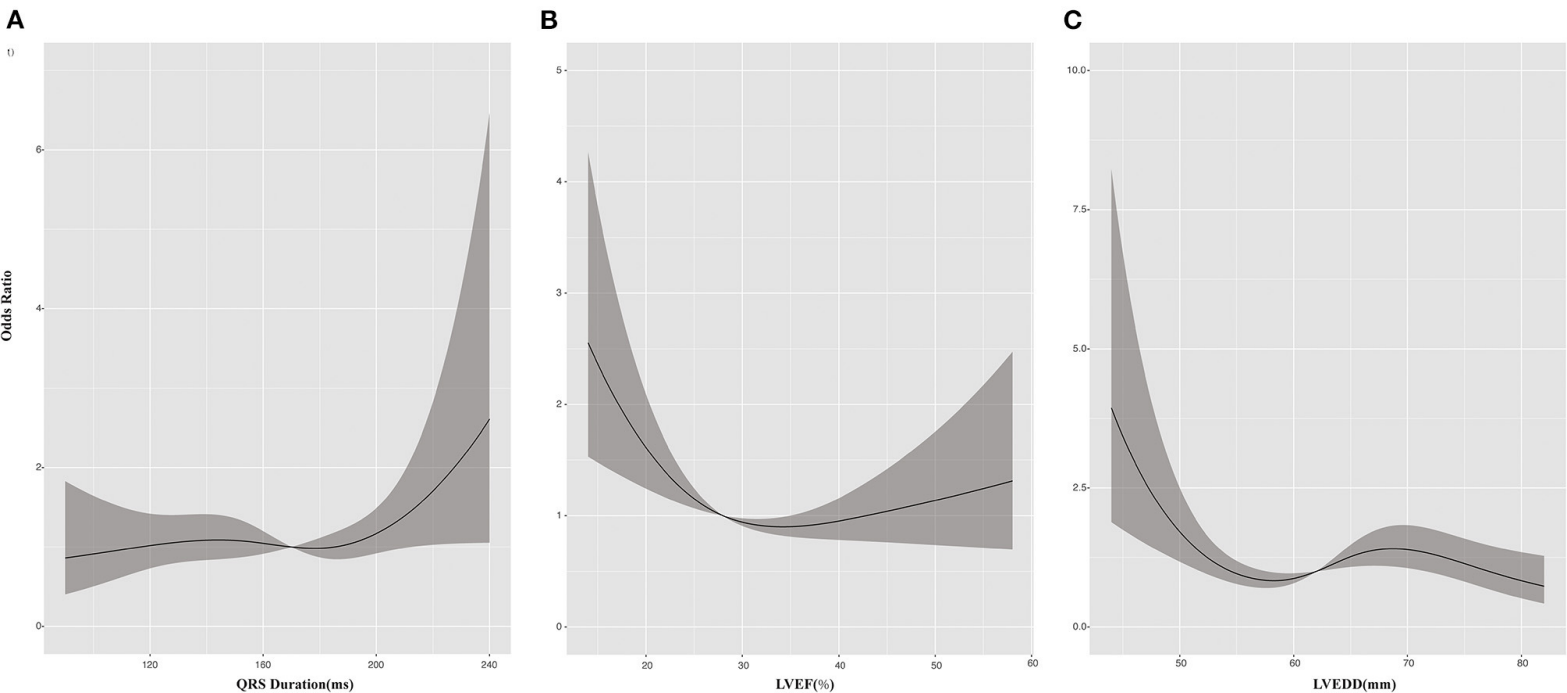
Cubic spline plots for predictors of response to cardiac resynchronization therapy (CRT). Plots show odds ratios (solid lines) and 95% CIs (gray shading) for response to CRT by **(A)** QRS duration, **(B)** left ventricular ejection fraction (LVEF), and **(C)** left ventricular end-diastolic dimension (LVEDD).

At 6-month follow up, 364 patients (47.8%) had a response to CRT, with an absolute LVEF increase of more than 5%. The rate of CRT response was 50.3% for patients with CRT upgrade, 55.8% for patients undergoing AVN ablation, 39.2% for patients with RBBB, and 36.8% for patients with IVCD. Multivariable logistic regression analysis suggested that preexisting high-degree atrioventricular block (including second- and third-degree atrioventricular block), wider QRS duration, and lower LVEF and a lower LVEDD were associated with greater CRT response, whereas chronic kidney disease (CKD) and RBBB were associated with poor response to CRT (Table 2). Figure 1 depicts the relationship between QRS duration, LVEF, and LVEDD and odds of CRT response. Each was adjusted for all the factors (age, sex, body mass index, hypertension, coronary artery disease, diabetes, CKD, ischemic cardiomyopathy, atrial fibrillation, high-degree atrioventricular block, QRS duration, LVEF, and LVEDD).

**Table 2:** Logistic regression model showing clinical predictors of CRT response.

	**Univariable**		**Multivariable**	
**Variable**	**OR (95%CI)**	***P-*value**	**OR (95%CI)**	***P-*value**
Age^a^	1.01 (1.00–1.03)	0.17		
Male sex	1.07 (0.70–1.65)	0.76		
Body mass index^a^	0.98 (0.95–1.01)	0.20		
Hypertension	1.06 (0.75–1.50)	0.74		
Coronary artery disease	0.90 (0.64–1.27)	0.55		
Diabetes	0.81 (0.54–1.22)	0.32		
Chronic kidney disease	0.77 (0.53–1.12)	0.18	0.68 (0.48–0.96)	0.03
Ischemic cardiomyopathy	0.80 (0.57–1.14)	0.21		
Atrial fibrillation	1.35 (0.95–1.93)	0.09		
High-degree atrioventricular block^b^	1.95 (1.36–2.79)	<0.001	1.61 (1.03–2.51)	0.04
QRS duration		0.007		0.01
<150 ms	Reference		Reference	
≥150 ms	1.70 (1.16–2.51)		1.66 (1.11–2.48)	
Paced ECG	1.76 (1.23–2.52)	0.002	0.69 (0.33–1.42)	0.32
RBBB	0.64 (0.38–1.07)	0.09	0.47 (0.29–0.76)	0.002
IVCD	0.63 (0.40–0.99)	0.045	0.71 (0.45–1.11)	0.14
LVEF^a^	0.98 (0.97–1.00)	0.07	0.94 (0.93–0.96)	<0.001
LVEDD^a^	0.96 (0.94–0.98)	<0.001	0.94 (0.92–0.96)	<0.001

*CRT, cardiac resynchronization therapy; ECG, electrocardiography; IVCD, intraventricular conduction delay; LVEDD, left ventricular end-diastolic dimension; LVEF, left ventricular ejection fraction; OR, odds ratio; RBBB, right bundle-branch block. Entire cohort (n = 761) was used for Logistic Regression Model*.

a *For all the continuous variables, the ORs were calculated by per 1-unit increase*.

b *High-degree atrioventricular block was defined as second- and third-degree atrioventricular block*.

**Figure 2:**
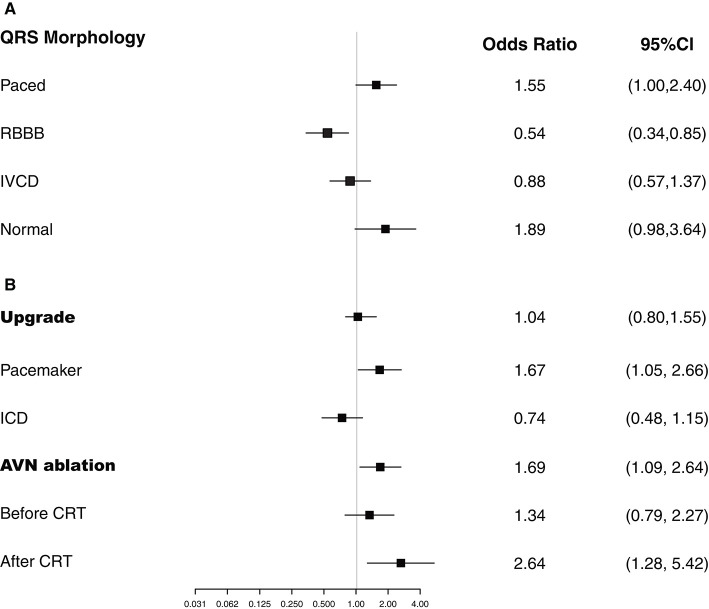
Logistic regression analyses. Plots show the effect of various predictors on response to cardiac resynchronization therapy (CRT). ICD, implantable cardioverter-defibrillator; IVCD, intraventricular conduction delays; RBBB, right bundle branch block.

Patients who had an upgrade from a pacemaker had a greater CRT response (OR, 1.67; 95% CI, 1.05–2.66), with LVEF improved from 32.4 to 39.7%, whereas those with CRT upgrade from an ICD had no significant benefit (OR, 0.74; 95% CI, 0.48–1.15), with a mean LVEF improvement from 25.9 to 29.2% (Figure 2). AVN ablation was associated with CRT response (OR, 1.69; 95% CI, 1.09–2.64), with mean LVEF improved from 32.3% to 40.1%, especially for patients who had AVN ablation at the time of CRT implant or after CRT (Figure 2).

### Predictors of Survival

**Figure 3:**
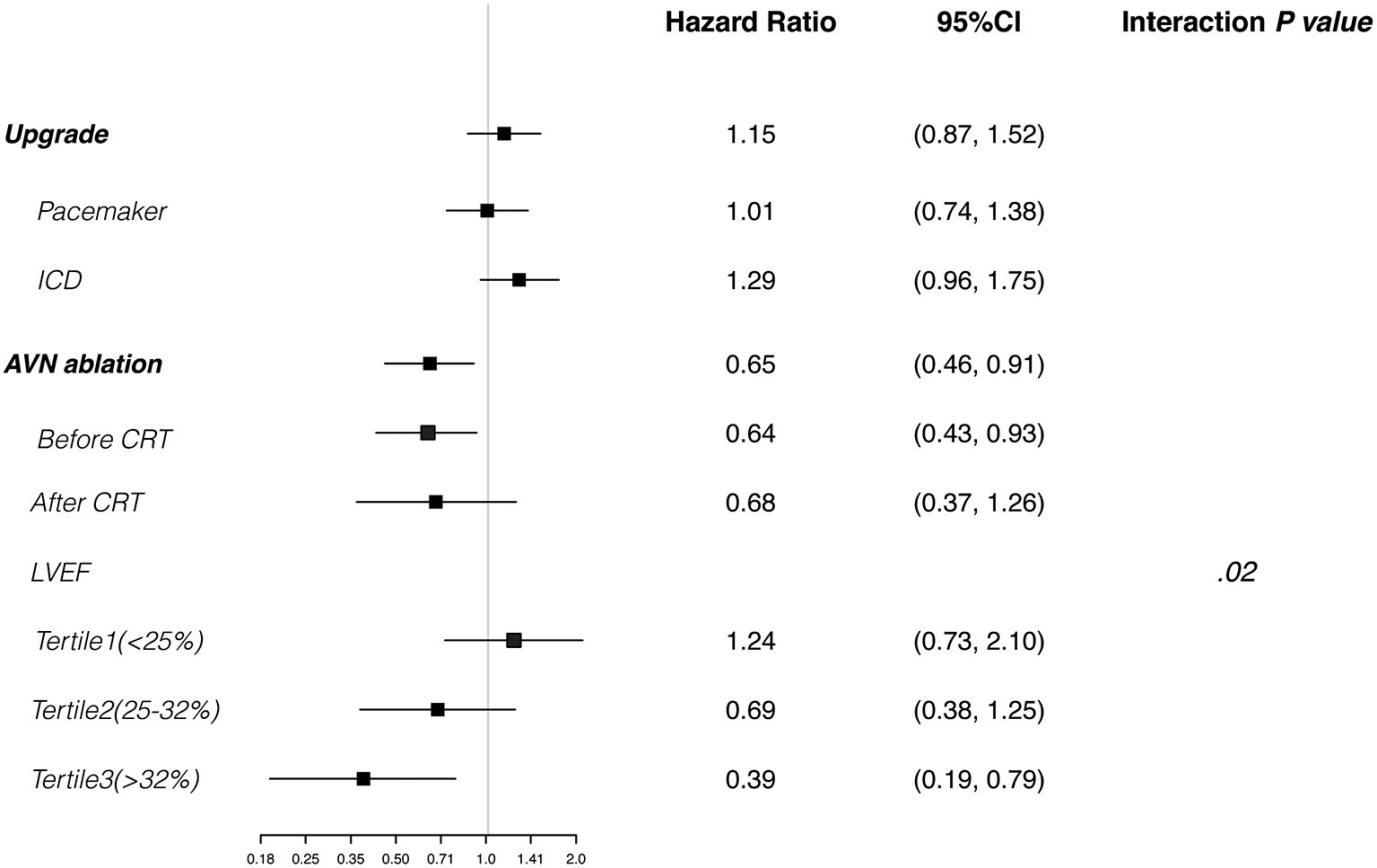
Cox regression analyses. Plots show the effect of various predictors on overall survival (hazard ratio for risk of all-cause death). CRT, cardiac resynchronization therapy; ICD, implantable cardioverter-defibrillator; LVEF, left ventricular ejection fraction.

The median follow-up time was 52 (interquartile range, 33–90) months. The 1-, 3-, and 5-year overall survival rates were 95.9, 82.3, and 66.7%, respectively. Multivariable Cox regression analysis suggested that older age, male sex, coronary artery disease, and higher creatinine value were independent predictors of all-cause death (Table 3). Use of a renin-angiotensin system inhibitor and high hemoglobin value were beneficial factors for survival. CRT upgrade had no significant benefit for overall survival (HR, 1.15; 95% CI, 0.87–1.52) compared with de novo implant (Figure 3). AVN ablation was associated with a lower mortality rate (HR, 0.65; 95% CI, 0.46–0.91), especially for those with a higher LVEF (Figure 3).

**Table 3:** Cox proportional hazards regression model showing clinical predictors of all-cause death.

	**Univariable**		**Multivariable**	
**Variable**	**HR (95%CI)**	***P-*value**	**HR (95%CI)**	***P-*value**
Age^a^	1.03 (1.02–1.04)	<0.001	1.02 (1.01–1.04)	<0.001
Male sex	1.50 (1.12–1.99)	0.006	1.54 (1.11,2.13)	0.008
Body mass index^a^	1.01 (0.99–1.03)	0.24		
Hypertension	1.15 (0.93–1.42)	0.19		
Coronary artery disease	1.86 (1.50–2.31)	<0.001	1.37 (1.03–1.83)	0.03
Ischemic cardiomyopathy	1.42 (1.15–1.76)	0.001		
Atrial fibrillation	1.15 (0.93–1.42)	0.20		
High-degree atrioventricular block^b^	0.80 (0.64–1.00)	0.054		
QRS duration		0.44		
<150 ms				
≥150 ms	0.91 (0.72–1.16)			
LVEF^a^	0.99 (0.98–1.00)	0.21		
LVEDD^a^	1.00 (0.98–1.01)	0.52		
β-Blocker	1.25 (0.92–1.70)	0.15		
RAS inhibitor	0.67 (0.53–0.84)	<0.001	0.59 (0.46–0.76)	<0.001
Spironolactone	1.16 (0.92–1.47)	0.20		
Hemoglobin^a^	0.87 (0.83–0.92)	<0.001	0.88 (0.83–0.94)	<0.001
Creatinine^a^	1.47 (1.32–1.64)	<0.001	1.25 (1.08–1.44)	0.002

*HR, hazard ratio; LVEDD, left ventricular end-diastolic dimension; LVEF, left ventricular ejection fraction; RAS, renin-angiotensin system. Entire cohort (n = 761) was used for Cox Proportional Hazards Regression Model*.

a *For all the continuous variables, the ORs were calculated by per 1-unit increase*.

b *High-degree atrioventricular block was defined as second- and third-degree atrioventricular block*.

**Figure 4:**
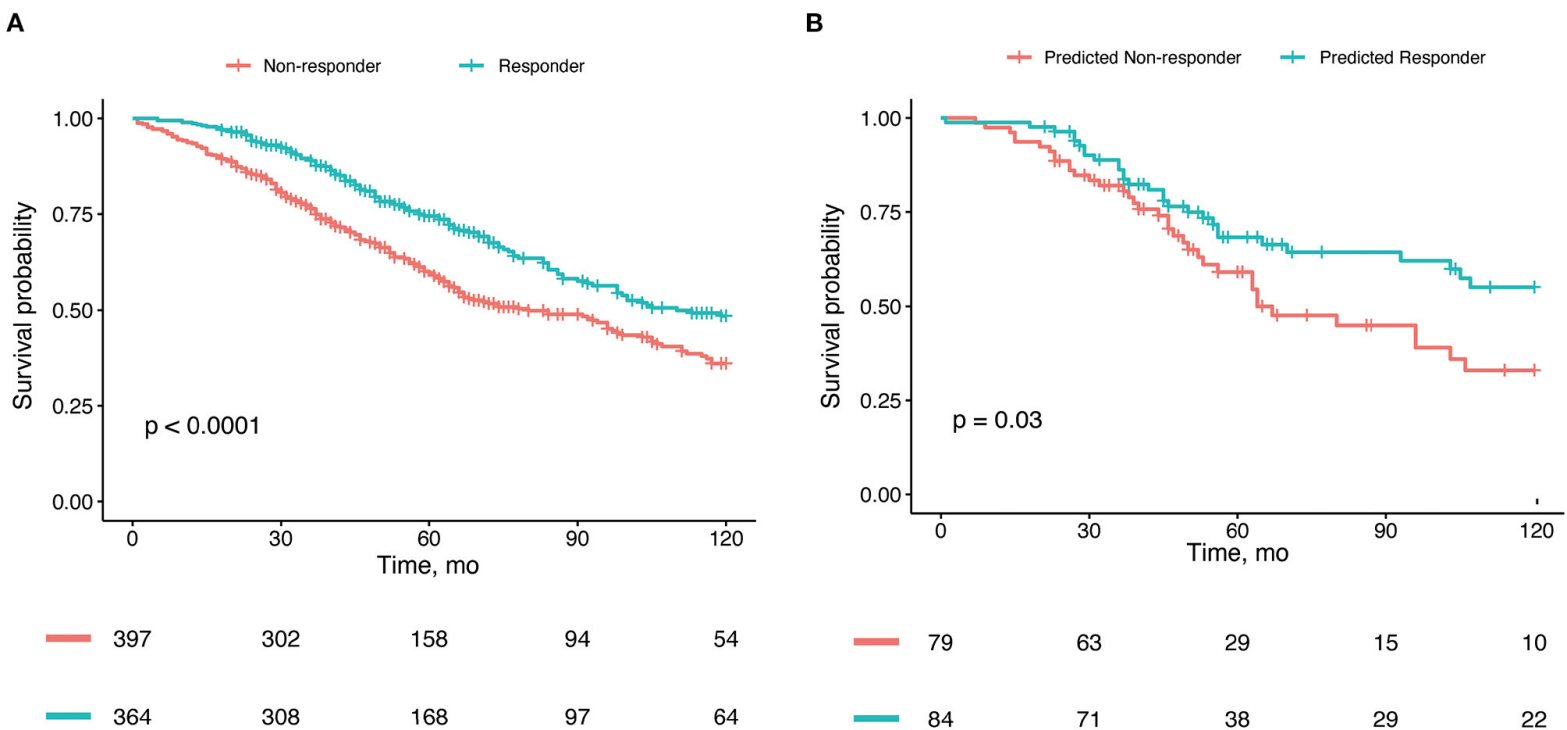
Kaplan-Meier estimates of overall survival. **(A)** Survival stratified by real response to cardiac resynchronization therapy (CRT) in the whole cohort. **(B)** Survival stratified by CRT response predicted by our nomogram model in the validation cohort.

Patients who were CRT responders had significantly better survival than nonresponders (HR, 0.68; 95% CI, 0.55–0.83; *P* < 0.001) (Figure 4A). The 5-year overall survival rate was 74.5% for responders and 59.7% for non-responders. After adjustment for the confounding factors, the HR for CRT responders was 0.63 (95% CI, 0.50–0.79; *P* < 0.001).

### CRT Response Nomogram Model

**Figure 5:**
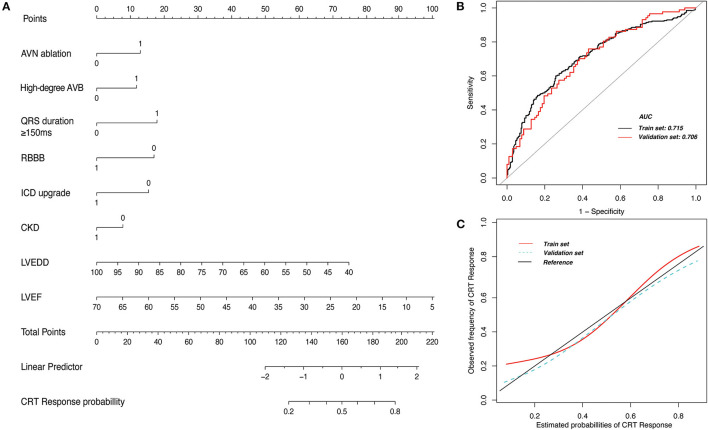
Nomogram prediction model for response to cardiac resynchronization therapy (CRT). **(A)** The nomogram model includes 8 clinical variables that were scored. The probability of CRT response is predicted by calculating the total points. **(B)** The area under the receiver operating characteristic curve (AUC) suggests good accuracy for estimating CRT response in the training set and the validation set. **(C)** The calibration plot suggests good consistency between risk estimation by the nomogram and the observed probabilities in the training set and the validation set. AVB, atrioventricular block; AVN, atrioventricular node; CKD, chronic kidney disease; ICD, implantable cardioverter-defibrillator; LVEDD, left ventricular end-diastolic dimension; LVEF, left ventricular ejection fraction; RBBB, right bundle-branch block. 1 and 0 were allocated for variables where 1 indicated presence of the variable and 0 indicated absence.

For prediction model development and performance evaluation, 549 participants were randomly assigned to the training cohort, and 212 participants were assigned to the validation cohort. Of the candidate baseline variables listed in Table 1; 8 variables (CKD, QRS >150 ms, high-degree atrioventricular block, RBBB, LVEF, LVEDD, AVN ablation, and ICD upgrade) were automatically selected with backward stepwise selection using the Akaike information criterion method for developing a prediction model for CRT response according to the linear regression model. The nomogram predicting CRT response based on this linear regression model is shown in Figure 5A. The nomogram demonstrated an area under the receiver operating characteristic curve of 0.71 (95% CI, 0.63–0.78) in the validation set (Figure 5B). The calibration plot suggested good consistency between the risk estimation by nomogram and the observed probabilities (Figure 5C). For example, if a patient who had a baseline QRS duration longer than 150 ms (18 points), LVEF of 35% (57.5 points), LVEDD of 60 mm (50 points) without RBBB (17.5 points) and CKD (10 points) underwent pacemaker upgrade (16 points) to CRT and AVN ablation (12.5 points), the patient would have a total of 181.5 points, which equates to a CRT response probability of about 70%.

The patients who were predicted by our nomogram model to be CRT responders had lower all-cause mortality rates than did those who were predicted to be CRT non-responders in the validation set cohort (HR, 0.54; 95% CI, 0.30–0.96) (Figure 4B).

## Discussion

In the current study, we developed a nomogram-based prediction model for CRT response that uses clinical variables in patients without intrinsic LBBB undergoing CRT. The principal findings were that (1) 70% of patients without intrinsic LBBB were undergoing CRT upgrade, and upgrade from a pacemaker (not an ICD), preexisting high-degree atrioventricular block with right ventricular pacing, and a wider QRS complex were associated with a greater CRT response; (2) the patients underwent AVN ablation was associated with greater CRT response and survival; and (3) our nomogram based on clinical features had optimal performance in predicting CRT response in patients without intrinsic LBBB undergoing CRT in our internal validation.

### CRT Response and Survival in Patients Without Intrinsic LBBB

The benefit of CRT in patients with LBBB has been proved in multiple RCTs; however, the use of CRT in patients without intrinsic LBBB has been considered to be less favorable. Current guidelines recommend a class IIa (QRS >150 ms) or IIb indication (QRS 120–150 ms) for CRT for patients without intrinsic LBBB (7–9). *Post-hoc* analysis of the COMPANION and MADIT-CRT studies, however, showed a benefit of CRT-ICD in prolonged PR interval patients who was without intrinsic LBBB (24, 25). Several studies emphasized that these patients without intrinsic LBBB, but with mechanical dyssynchrony, had improved survival after CRT (26, 27). We found that lower LVEF and longer QRS duration were associated with a higher probability of CRT response for patients without intrinsic LBBB, which suggests that QRS duration of 150 ms or longer was an important predictor of CRT response for patients without intrinsic LBBB. A recent prospective study showed that CRT-ICD was associated with better outcomes in patients with IVCD and QRS longer than 150 ms but not in patients with RBBB (28). Other studies have also shown that RBBB was associated with worse outcomes (29, 30), although left ventricular dyssynchrony may be present concomitantly in patients with RBBB (31). Our study confirmed previous findings that neither RBBB nor IVCD was favorable for CRT response. It is not surprising that upgrade from pacemaker (vs. ICD) was associated with better response to CRT. The ICD patients likely had pre-existing cardiomyopathy, whereas the pacemaker patients likely developed cardiomyopathy due to RV pacing (functionally acquired LBBB physiology).

AVN ablation decreases mortality rates in patients with permanent atrial fibrillation (32, 33). CRT at the time of AVN ablation is optimal to ensure biventricular pacing for patients with permanent atrial fibrillation and decreased LVEF (34). Our study patients who underwent AVN ablation had significantly higher CRT response rates than did those without AVN ablation. Because both AVN ablation and upgrade from right ventricular pacing to CRT were favorable factors, the likelihood of CRT response was attributed to the correction of mechanical dyssynchrony and well-controlled ventricular rate, which could be detrimental components of LVEF (13). In agreement with previous studies, age, male sex, coronary artery disease, lower hemoglobin values, and higher creatinine levels predicted poor survival in our study (35).

### Nomogram Model to Assess CRT Response

Nomograms have been developed for cancer and cardiovascular diseases to facilitate therapy selection or determine the prognosis (20). Owing to the complexity of CRT response in patients without intrinsic LBBB, a predictive model with representative clinical features is of value. Therefore, we developed a nomogram model with common clinical features for the prediction of CRT response. We found that the nomogram model can leverage clinical variables to assess patients without intrinsic LBBB who may be likely or unlikely to benefit from CRT; these patients, in general, have a low response to CRT. The favorable factors were AVN ablation, preexisting atrioventricular block requiring pacing, wider QRS duration, a less dilated left ventricle, and lower LVEF, whereas RBBB, CKD, and the presence of an ICD were unfavorable factors. The predicted percentage of CRT response can be estimated from these factors. However, because our nomagram model was only based on single-center database, we will consider a prospective study extend to other center.

### Limitations

This study had some limitations. All benefits from CRT were relative to those in other participants without LBBB morphology. To minimize the effects of population differences, all results were adjusted for baseline characteristics. Some data also were missing and could not be obtained from the database or chart review. Due to a long-time span in our study, some heterogeneity in the technology of CRT may affect the explanation of these results. In addition, some important variables such as N-terminal pro–brain natriuretic peptide and echocardiography parameters were excluded because of more than 10% missing values. Because this was a retrospective study, patient selection bias may exist. Finally, we only verified the nomogram model with internal validation in our single-center CRT database, and external validation was not performed. A new prospective study is needed to further validate our nomagram model.

### Conclusion

Patients who underwent CRT upgrade from a pacemaker and AVN ablation had better CRT response and survival. A nomogram model was developed for the patients without intrinsic LBBB to assess CRT response rate and further facilitate shared decision-making in selecting CRT candidates without intrinsic LBBB.

## Data Availability Statement

The original contributions presented in the study are included in the article/supplementary material, further inquiries can be directed to the corresponding author/s.

## Ethics Statement

The studies involving human participants were reviewed and approved by Mayo Clinic Institutional Review Board. The patients/participants provided their written informed consent to participate in this study.

## Author Contributions

All authors listed have made a substantial, direct and intellectual contribution to the work, and approved it for publication.

## Funding

Research funding from the National Natural Science Foundation of China (Grant No. 81600215 to P-LX) and Kuanren Talents Program of the second affiliated hospital of Chongqing Medical University (to P-LX).

## Conflict of Interest

The authors declare that the research was conducted in the absence of any commercial or financial relationships that could be construed as a potential conflict of interest.

## Publisher's Note

All claims expressed in this article are solely those of the authors and do not necessarily represent those of their affiliated organizations, or those of the publisher, the editors and the reviewers. Any product that may be evaluated in this article, or claim that may be made by its manufacturer, is not guaranteed or endorsed by the publisher.

## Abbreviations

AVN: atrioventricular node
CKD: chronic kidney disease
CRT: cardiac resynchronization therapy
HF: heart failure
ICD: implantable cardioverter-defibrillator
LBBB: left bundle-branch block
LVEF: left ventricular ejection fraction
LVEDD: left ventricular end-diastolic dimension
IVCD: intraventricular conduction delay
RBBB: right bundle-branch block.
